# AKR1B10 in gastrointestinal diseases

**DOI:** 10.18632/aging.100737

**Published:** 2015-04-10

**Authors:** Yi Shen, Duan-Fang Liao, Deliang Cao

**Affiliations:** Department of Medical Microbiology, Immunology, & Cell Biology, Simmons Cancer Institute, Southern Illinois University School of Medicine, Springfield, IL, USA 62794

Ulcerative colitis (UC) is characterized by long-standing mucosal inflammation of the large bowel, with increased tendency of developing colorectal cancer (CRC), i.e., colitis-associated cancer (CAC) [1]. The other two predisposing conditions for CRC are familial adenomatous polyposis (FAP) and hereditary nonpolyposis colorectal cancer (HNPCC), whose genetic etiology is well defined [1]. The genetic predisposition of UC has not been firmly delineated thus far. The recent work from our laboratory characterized aldo-keto reductase 1B10 (AKR1B10) as a novel risky factor of UC and CAC [2].

Almost all CRCs progress from a dysplastic precursor lesion, but the development of CAC is different in several important aspects from sporadic CRC [3]. Dysplasia in patients with sporadic CRC is usually the adenomatous polyp (adenoma), a discrete neoplastic focus; in contrast, CAC develops from dysplastic lesions that can be polyploid, flat, localized, or multifocal. In addition, the molecular abnormalities of inflamed colonic mucosa in CAC appear present much earlier than any histological evidence (dysplasia or cancer). This raises an important question of how chronic inflammation leads to the neoplastic transformation and CRC pathogenesis. Inflammatory oxidative stress likely plays a causative role. Reactive oxygen species target a wide range of macromolecules, including proteins, DNA and lipids, and induce cellular damage that may be associated with epithelial homeostasis [4]. Under the inflammatory environment, free radicals and other prooxidant molecules generated by neutrophils and macrophages can also inflict lipid peroxidation and biomembrane damage [4]. Lipid peroxides are electrophilic carbonyl compounds and are highly cytotoxic and genotoxic. They may serve as secondary contributors to cellular and DNA damage may target key genes or proteins responsible for dysplasia and subsequent arises of carcinoma [5]. AKR1B10 is primarily expressed in epithelial cells of gastrointestinal tract, and exerts a protective role through eliminating oxidative and carbonyl stresses and promoting epithelial proliferation for damage repair in inflammation. However, AKR1B10 expression is lost or markedly decreased in over 90% UC and CAC [2]. AKR1B8 in the mouse is the ortholog of human AKR1B10. To mimic the phenomenon seen in humans, we disrupted the AKR1B8 locus in mice. Initial findings showed that AKR1B8 deficiency diminished proliferation, migration, and maturation of colonic crypt cells, disrupting the epithelial homeostasis. As a result, the AKR1B8 deficient mice were susceptible to dextran sodium sulfate (DSS)-induced colitis and demonstrated delayed re-epithelialization and epithelial remodeling, leading to more severe inflammatory and neoplastic lesions. In the setting of heightened epithelial corruption, mutagenic assaults and sustained DNA damage caused by oxidative and carbonyl stresses in the AKR1B8 deficient epithelium appear to drive the carcinogenic procession. As such, the process of colitis-neoplasia in the AKR1B8 deficient mice behaves similarly to aging that accumulates DNA damage that fails to repair.

Oxidative stress is a leading theory of aging. Progressive oxyradical overloads with aging leading to age-associated physiological function declines [6]. Data from antioxidant studies suggest although the rate of oxidative damage decreases with aging, possibly due to the reducing rate of metabolism, the steady-state levels of oxidative DNA modifications increase due to insufficient repairing [7]. Similarly, oxidative and carbonyl-associated DNA damage/mutations, such as G:C to A:T, are accumulated in AKR1B8 deficient mice. A genome-wide sequencing analysis revealed colitis-associated DNA mutations in up to 28 oncogenes or tumor suppressors uniquely in AKR1B8 deficient colon mucosa. This indicates failure of repairing oxidative damage, such as lipid peroxidation, in AKR1B8 deficient colon, supporting the protective role of AKR1B10 in human colon.

It is becoming clear that AKR1B8 deficiency favors tumorigenesis due to increased accumulation of DNA mutations in host cells. At the same time, it is reasonable to speculate that AKR1B8 deficiency may also associate with certain signaling pathways that regulate cell proliferation and survival. Indeed, our work demonstrated that AKR1B8 mediates *de novo* lipid synthesis [2], which may affect lipid second messenger-mediated cell signaling transducers, such as PI3K/AKT and PKC/ERK. Overall, our studies suggest that AKR1B10 is an important protector in the gastrointestinal epithelium. AKR1B10 deficiency may be a new predisposition of UC and CAC. Human risk of developing gastrointestinal diseases increases with age. It would be interesting to see if AKR1B10 expression in the epithelium declines with aging.

## References

[1] Xavier RJ, Podolsky DK (2007). Nature.

[2] Shen Y (2015). Clinical cancer research.

[3] Itzkowitz SH, Yio X (2004). American journal of physiology Gastrointestinal and liver physiology.

[4] Hussain SP (2003). Nature reviews Cancer.

[5] Niki E (2005). Biochemical and biophysical research communications.

[6] Harman D (1956). Journal of gerontology.

[7] Loft S, Poulsen HE (1996). Journal of molecular medicine.

